# *Bacillus tropicus* and *Bacillus safensis* : A Potential Siderophore Producing Rhizospheric Bacteria: Its Characterisation and Optimisation

**DOI:** 10.1111/1758-2229.70388

**Published:** 2026-10-16

**Authors:** Anita Tilwari, Sanober Kalam

**Affiliations:** ^1^ Department of Microbiology Barkatullah University Bhopal Madhya Pradesh India

**Keywords:** *bacillus sp*, chelators, iron, optimisation, siderophore

## Abstract

Siderophore‐Producing Rhizobacteria (SPR) have emerged as a viable approach to plant protection and crop health management in agriculture. It brings iron molecules into bacterial cells for use in a variety of metabolic processes, with applications in agriculture, medicine, industry, and the environment. In this study, 22 bacteria were isolated and screened for siderophore production from maize field soil. Seven of the 22 bacterial isolates showed discrete zones of orange colour, indicating siderophore production. However, only two potent siderophore‐producing strains, *Bacillus safensis* (BMS—01) and *Bacillus tropicus* (BMS—15), were optimised and identified using physiological characteristics and 16S rDNA sequencing. After optimising siderophore production with glucose and sucrose as carbon sources and NaNO_3_ and (NH_4_)_2_NO_3_ as nitrogen sources, *Bacillus safensis* (BMS—01) and *Bacillus tropicus* (BMS—15) were found to have a significant ability to yield siderophore (60%–70% units) under ideal conditions of pH 8, 28°C. The study concludes that the strains *Bacillus safensis* and *Bacillus tropicus* show potential for siderophore production, which may warrant further investigation as biocontrol agents and plant growth promoters in future field trials.

## Graphical abstract

Rhizospheric bacteria were isolated from maize field soil and screened for siderophore production using the Chrome Azurol S (CAS) assay. Among 22 bacterial isolates, two potent strains, *Bacillus safensis* (BMS—01) and *Bacillus tropicus* (BMS—15), exhibited the highest siderophore production. Molecular identification using 16S rDNA sequencing confirmed the identity of both strains. Optimisation of culture conditions revealed maximum siderophore production (60%–70% siderophore units) at pH 8, 28°C, with glucose/sucrose as carbon sources and NaNO_3_/(NH_4_)_2_NO_3_ as nitrogen sources. These promising isolates may be further developed as plant growth‐promoting rhizobacteria and environmentally friendly biocontrol agents for sustainable agricultural applications following comprehensive field evaluation.

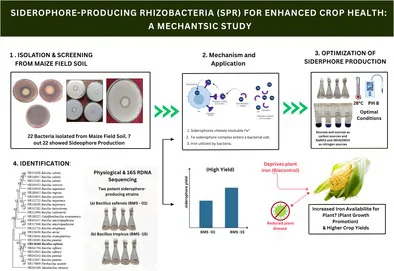

## 1 Introduction

In addition to macronutrients, plants require iron for proper growth. While some strains of Lactobacilli may not require iron, the majority of microbes do. Microbes have less iron to work with when exposed to free metal iron (Fe(III)) in aerobic environments because it forms insoluble hydroxides and oxyhydroxides. Bacteria have a way of absorbing iron when these conditions exist; they dissolve the metal form of iron. The conventional approach involves creating chelators with low molecular weight that exhibit strong iron complexing capabilities (J. B. Neilands 1995; Miethke and Marahiel 2007). Al, Cd, Cu, Ga, In, Pb, and Zn are just some of the metals that these chelators can form stable complexes with (Christenson and Schijf 2011; Neubauer et al. 2000). A group of about 500 biomolecules called “siderophores” is produced, transported, and reimported into cells by different genes and regulators. Hydroxamate, catecholate, or mixed hydroxyl carboxylic ligand groups are the structural subtypes of these siderophores (Challis 2005; Visca et al. 2007). Even when iron isn't present, gram‐positive and gram‐negative bacteria can still create complexes with iron from their surroundings by producing siderophores (Saharan and Nehra 2011; Tian et al. 2009; Boukhalfa and Crumbliss 2002). To accomplish their job, siderophores first attach to ferric iron, creating a complex that can then enter cells through membrane‐bound siderophore receptors. The transport of the siderophore‐iron complex is made possible in gram‐positive bacteria by the involvement of siderophore irreversible proteins, permeases, and ATPases (Calvente et al. 2001 and Saharan and Nehra 2011).

Siderophores and their chemically modified derivatives have gained significant attention due to their wide‐ranging applications in medicine, agriculture, and environmental sciences. In agricultural systems, they play a dual role by suppressing phytopathogenic microbes and at the same time promoting plant growth (Molnár et al. 2023). Their strong metal‐chelating ability also makes them effective in the remediation of heavy‐metal‐polluted environments (Gupta et al. 2023). Beyond this, siderophores are increasingly explored as natural biosensors, particularly for monitoring iron availability in different ecological niches (Khasheii et al. 2021).

In the medical field, their importance is even more pronounced. By employing the so‐called “Trojan horse strategy,” siderophores can be linked with antibiotics, ensuring targeted delivery to drug‐resistant bacterial strains (Rayner et al. 2023). They also show therapeutic potential in managing iron overload disorders, including conditions such as sickle cell anaemia (Ribeiro et al. 2022). Additional studies highlight their promise in antimalarial treatments, removal of toxic transuranic elements from the body, and even as candidates with anticancer properties (Pita‐Grisanti et al. 2022).

Because of their intricate membrane structure, gram‐negative bacteria have a distinct mode of transport. An ATP‐binding cassette transporter (ABC‐transporter)–like cytoplasmic membrane protein and a periplasmic binding protein are employed to transfer the siderophore‐iron complex in this instance. Upon entering the cytosol, the siderophore chelator complex is immediately disassembled, resulting in the reduction of ferric iron to ferrous form and its subsequent release. This freed ferrous iron state is also utilised in their metabolic processes. Depending on the efflux pump system, the siderophore in its free form is either oxidised or recycled (Ahmed and Holmström 2014).

But a siderophore's principal role is to facilitate microbial multiplication by providing soluble iron. In addition to their many uses in biosensors, agriculture, bioremediation, and medicine, they have many other potential uses. Siderophore‐producing bacteria have been reported to exhibit significant antagonistic activity against diverse plant pathogenic fungi. Under the optimised culture conditions described in this study, which counts siderophore production, the isolates produce a higher concentration of these compounds.

## 2 Materials and Methods

### 2.1 Isolates Isolation and Identification

The soil specimen was obtained from the rhizospheric area of the maize crop field housed at Barkatullah University's Department of Microbiology in Bhopal, Madhya Pradesh. The chosen plants were delicately uprooted, and any loosely bound soil was stripped away by meticulous agitation of the roots. Extraction of soil firmly attached to the roots was done by brushing it off the roots and placing it into a plastic tray or bag. The rhizosphere soils obtained from maize were gathered, packed into polyethylene bags, and promptly transported to the laboratory under a cooler to prevent drying out. A soil suspension was made by dissolving 1 g of soil sample in 9 mL of sterile distilled water. To obtain pure colonies, the soil suspension was spread on nutrient agar plates and incubated at 28°C ± 2°C for 24–48 h. Successfully isolated colonies were carefully chosen, refined, and kept on nutrient agar slants (Gaonkar and Bhosle 2013).

### 2.2 Siderophore Estimation (Quantitative and Qualitative)

The CAS agar test was employed for the qualitative estimation of siderophore. This involved inoculating the Nutrient agar medium with the bacterial isolate, adding the prepared CAS solution, and then incubating the mixture for two weeks in dark conditions at 28°C. The presence of orange zones served as confirmation of the siderophore production. A 100 mL CAS solution was prepared by dispersing 60.5 mg of CAS in 50 mL of deionised water and then adding 10 mL of a solution of FeCl_3_.6H_2_O. A solution of 72.9 mg of hexa‐decyl trimethyl ammonium bromide (HDTMA) in 40 mL of deionised water was added to 100 mL of CAS fluid.

The bacterial isolates were cultured in an iron‐deficient succinate medium (Composition: 6.0 g/L K_2_HPO₄, 3.0 g/L KH_2_PO₄, 1.0 g/L (NH₄)_2_SO₄, 0.2 g/L MgSO₄·7H_2_O, and 4.0 g/L succinic acid; pH 7.0). Iron‐deficient conditions were maintained by treating all glassware with 6 M HCl and rinsing thoroughly with deionised water to reduce contaminating iron to < 1 μM and incubated for 48 h while being continuously shaken at 120 rpm for the quantitative estimation. To test each isolate's potential to produce siderophores, a spectrophotometric technique was employed. The number of siderophores produced by the isolate was measured using the Chrome Azurol Sulphonate (CAS) assay, as described by Schwan and Neiland. A 100 mL CAS solution was prepared by dispersing 60.5 mg of CAS in 50 mL of deionised water and then adding 10 mL of a solution of FeCl_3_.6H_2_O. A solution of 72.9 mg of hexa‐decyl trimethyl ammonium bromide (HDTMA) in 40 mL of deionised water was added to 100 mL of CAS fluid.

Fifty millilitres of the newly made CAS solution, along with fifty millilitres of culture supernatant, should be incubated for five minutes. In order to synthesise the siderophore, the mixture was then measured at 630 nm. The intended method for expressing the percentage of siderophore in terms of the percentage of siderophore units was calculated relative to a non‐inoculated medium control (reference) using the following formula:

$$\%\text{of siderophore units}=\left\{\left(\mathrm{Ar}-\mathrm{As}\right)/\mathrm{Ar}\right\}\times 100$$

where Ar is the absorbance of the reference (CAS solution + uninoculated medium) and As is the absorbance of the sample (CAS solution + culture supernatant) at 630 nm.

All experiments were performed in triplicates (B. Neilands 1987; Chaiharn et al. 2009).

### 2.3 Characterisation of Efficient Siderophore‐Producing Isolate

Additional research was conducted to investigate the morphological, biochemical, and molecular characteristics of a bacterial strain that is capable of producing siderophores in an effective manner. In order to determine the shape, size, organisation, and gram nature of the isolated substances, they were stained. The isolated samples that had been purified were then subjected to additional analysis for the purpose of biochemical characterisation and the identification of the organism genus. Additionally, molecular characterisation was performed by sequencing the amplicon of the PCR product in both the forward and reverse directions. It was determined that the consensus sequence for the 16S rDNA gene, which is approximately 1200–1350 base pairs in length, was found. After that, a homology search was carried out on the sequences by utilising the NCBI BLAST program (Saharan and Nehra 2011).

### 2.4 Optimisation of Siderophore Production

The one‐variable‐at‐a‐time (OVAT) methodology was employed for preliminary screening of optimal conditions. Parameter ranges vary: pH (4–10), temperature (28°C–45°C), carbon sources (sucrose, glucose, fructose), nitrogen source (ammonium nitrate, sodium nitrate, urea), iron concentration (25, 50, 100, 150 μM), and organic acid (citric acid, acetic acid, oxalic acid) were selected based on the known physiological limits of rhizospheric *Bacillus* species. While OVAT allows for the identification of primary influencing factors, it is noted that this method does not account for complex interaction effects between variables, which could be further explored using Response Surface Methodology (RSM). Bacterial isolates were cultured under a range of fermentation conditions, including temperature, pH, nitrogen source, carbon source, iron concentration, and organic acid, in order to maximise siderophore synthesis. Bacteria were cultured in succinate medium for 48 h at different temperatures to produce the isolates. The siderophores were studied by centrifuging the supernatant at 5000 rpm for 10 min. We next used the CAS assay to check the cell‐free supernatant. Siderophore production was determined at 630 nm according to the method proposed by Fazary et al. (Fazary et al. 2016; Maindad et al. 2014).

### 2.5 Statistical Analysis

All experiments were performed in biological triplicates to ensure reproducibility. Data are presented as mean ± standard deviation (SD). Statistical significance was determined using ANOVA and *t*‐test, with a *p*‐value < 0.05 considered statistically significant. Analysis was conducted using Microsoft Excel.

## 3 Results and Discussion

### 3.1 Isolation and Screening of Siderophores Producing Bacteria

Because of their ability to bind ferric iron to ferric hydroxide complexes, certain organisms produce siderophores, which are chelating compounds with low molecular weight. Their contributions to improving plant growth, implementing biocontrol measures, and solving different environmental problems are substantial. This study sought to understand the culture medium that promotes high production of siderophores by isolating bacteria that produce them from soil samples and investigating the optimal fermentation conditions (Postle 1990 and Kannahi and Senbagam 2014).

More than 22 different bacterial groups were isolated and cultured from the soil sample taken from the root zone. The specific bacterial isolates that produced siderophore were quantitatively and qualitatively eliminated using the CAS assay. After the cultures were incubated in succinate broth medium for 48 h, the liquid at the top was carefully strained out and tested for CAS using spectrophotometry. The succinate medium was turbid for two of the twenty‐two bacterial isolates that tested positive for CAS. Two bacterial isolates were tested using the CAS agar plate method, which provided additional evidence that siderophores could be detected. The two strains were selected for further study because the bacterial isolates showed separate zones with an orange colour (Figure 1), which indicates that siderophore is being synthesised. The dye complex gradually changes colour from blue to orange when iron chelators, like siderophores, are added to the medium, removing iron from the mixture. The medium turns blue because iron chelators like CAS or HDTMA form a strong complex with the ferric ion (Sivasakthi et al. 2013; Louden et al. 2011).

**FIGURE 1:**
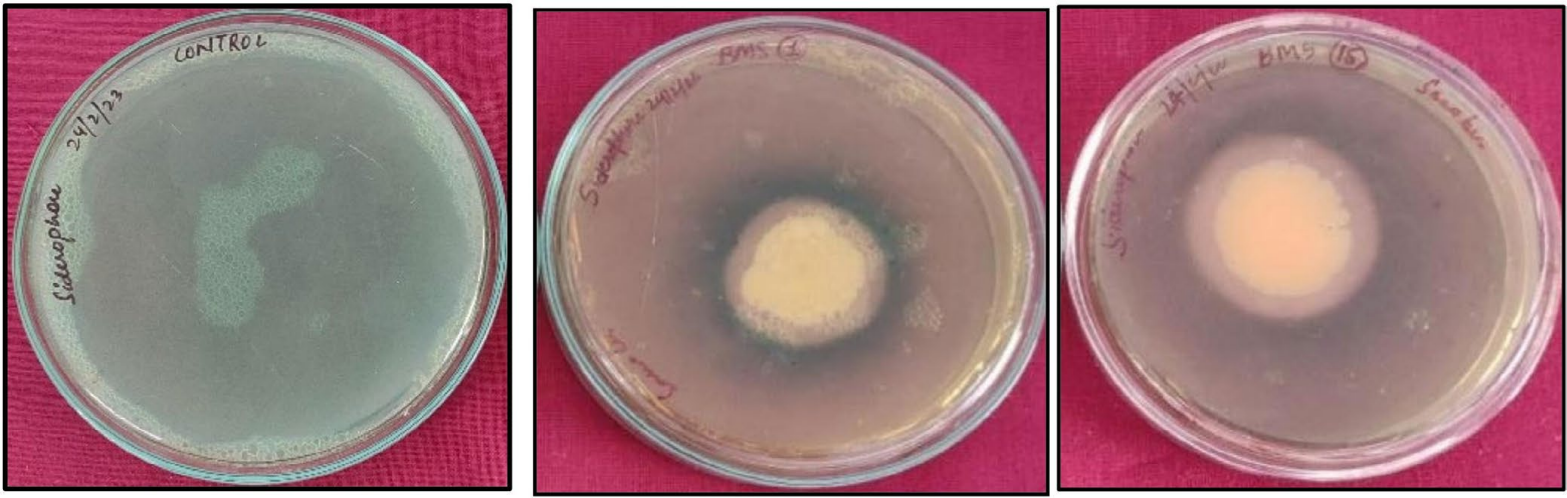
The emergence of an orange hue and the development of zones suggest the production of siderophores in the CAS agar plate assay (a) control (b, c) positive result.

### 3.2 Analysis of a Highly Effective Isolate That Produces Siderophores

The isolates BMS 01 and BMS 15 were additionally collected for morphological, molecular, and biochemical characterisation. Gram‐positive *Bacillus* sp. was indicated by the results of BMS 01 and BMS 15 g stain, which revealed rod shape with chains of continuously connected violet colour. Additionally, the results of gram staining have been validated by the biochemical characterisation of the bacterial isolates BMS 01 and BMS 15. Table 1 presents the outcomes of biochemical characterisation. Bacterial isolates BMS 01 and BMS 15 were identified via 16S rDNA sequencing (GenBank Accession Numbers: OR136465 and OR136385, respectively). The sequences showed 99% identity to *Bacillus safensis* and *Bacillus tropicus* over an alignment length of approximately 1218 and 1339 bp. Phylogenetic analysis with 1000 bootstrap replicates (Figure 2) confirmed their evolutionary placement within the *Bacillus* genus. The same reported for efficient siderophore production using *Bacillus* sp. were also published by Venkat et al.

**TABLE 1:** Analysis of Siderophore‐producing bacterial isolates.

Biochemical test	BMS 01	BMS 15
MR	+ve	+ve
VP	+ve	+ve
Indole	−ve	−ve
Citrate	−ve	−ve
Amylase	+ve	+ve
Oxidase	+ve	+ve
Catalase	−ve	+ve

**FIGURE 2:**
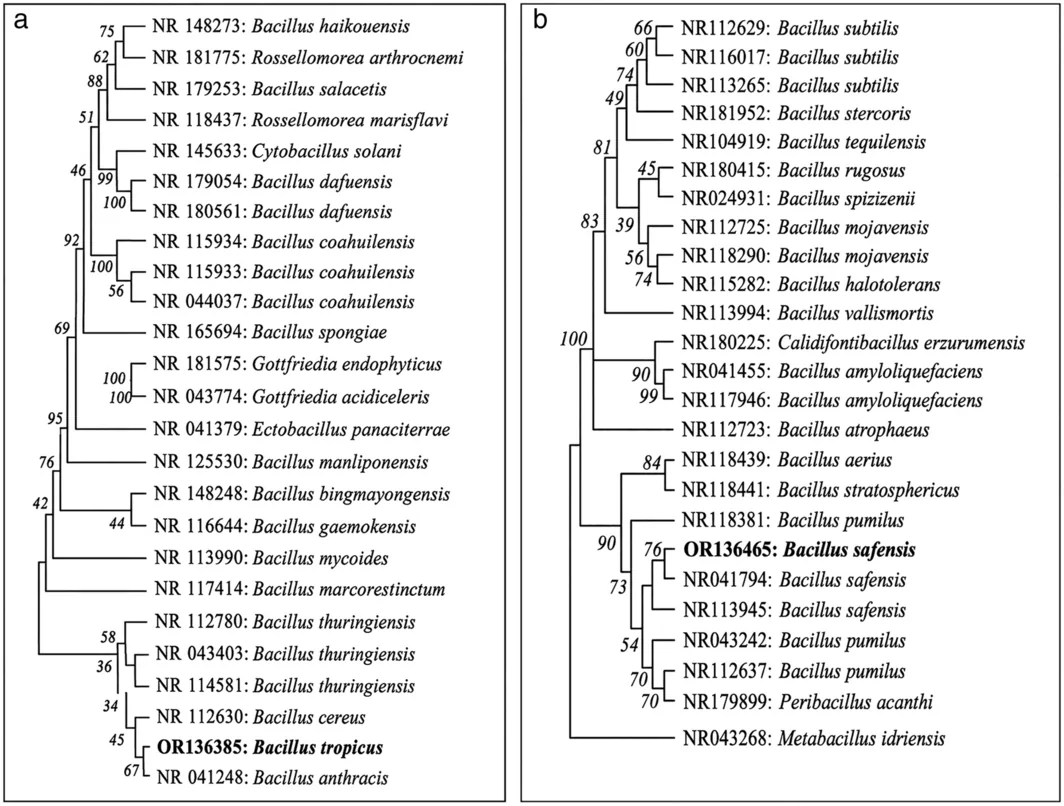
Phylogenetic tree of 16S rRNA genome of Bacteria isolate under study (OR136465 and OR136385) showing close relationships with *Bacillus safensis* and *Bacillus tropicus*. Phylogenetic tree perused using the (MEGA v.11.0) with 1000 replicate bootstrapping and dendrogram was generated with the neighbour joining method and viewed by the NJ plot program.

### 3.3 Quantitative Assessment of Siderophore Production From BMS 01 and BMS 15 Strain

A Chrome Azurol Sulphonate (CAS) assay was used for quantitative estimate, and the OD value was determined at 630 nm in accordance with Schwyn and Neiland's instructions. The aforementioned formula was used to calculate the proportion of the siderophore production under neutral pH conditions and room temperature. A variety of optimisation parameters were chosen after BMS 01 and BMS 15 estimates of 27.80% and 49.12% of siderophore production, respectively, quantitatively confirmed the generation of siderophores. In this study, the presence of siderophores was confirmed through the universal CAS assay; however, specific colorimetric classification via Arnow and Csaky tests was not concluded. It is important to note the inherent limitations of these traditional functional group‐specific assays (Moon et al. 2008).

First, the structural diversity of siderophores extends significantly beyond the classic catecholate and hydroxamate categories. Many microorganisms, particularly those in diverse ecological niches, produce carboxylate or phenolate‐type siderophores (such as rhizobactin or staphyloferrin) which do not react with Arnow or Csaky reagents, leading to potential false‐negative results despite strong iron‐chelating activity in the CAS assay (J. B. Neilands 1995).

Second, the sensitivity of these tests is highly dependent on the concentration of the siderophore in the supernatant. Previous studies have indicated that traditional assays often have higher detection thresholds compared to the CAS method, which can detect iron‐ligand complexes at lower molarities (Schwyn and Neilands 1987). Consequently, a negative result in a class‐specific test does not definitively indicate the absence of that chemical group but may instead reflect a concentration below the assay's analytical limit.

Furthermore, the chemical processing required—specifically the acid hydrolysis step in the Csaky test—can result in the degradation of certain labile or complex siderophore structures, thereby masking their presence. Given these constraints, the current study prioritises the functional validation of iron sequestration. Future investigations utilising advanced Liquid Chromatography‐Mass Spectrometry (LC–MS) or Nuclear Magnetic Resonance (NMR) spectroscopy are required to provide a definitive structural elucidation of the produced ligands.

### 3.4 Varieties of Culture Media for the Most Efficient Siderophores

In order to better understand how different culture conditions affect siderophores production and bacterial growth, optimisation was carried out using the One‐Variable‐at‐a‐Time (OVAT) methodology to identify the primary factors influencing siderophore yield. While OVAT provides a clear baseline for optimal pH and temperature, it is noted that this method does not account for interaction effects between variables, which may be addressed in future studies using Response Surface Methodology (RSM). Siderophores synthesis is heavily reliant on iron but is also greatly affected by a number of environmental factors in the culture medium. Considerations include varying pH levels, temperature swings, carbon and nitrogen sources, the presence of organic acids, and iron concentration (Shaikh et al. 2016).

#### 3.4.1 Effect of pH

The growth of microbes is greatly affected by pH. The availability and solubility of iron for growing microbes are affected by the acidity of the medium. Tailor and Joshi state that the peak synthesis of S‐11 siderophore occurs at pH 7, because iron remains insoluble at neutral pH and because bacterial growth is optimal in the physiological conditions of BMS 01 and BMS 15. The most significant production of iron chelators occurs at a pH of 8, according to our findings (Tailor and Joshi 2012). Our results are in agreement with those of Agro Services International, which states that iron becomes less soluble at higher pH values. Soil siderophores may be more readily produced when the pH drops below 8, which reduces iron solubility in soil solutions. Figure 3e shows that, at pH 8, the bacterial isolates produce more siderophores than the siderophore‐producing species *Rhizobacteria* and *Pseudomonas*, which are known to improve plant growth and stress resilience (Louden et al. 2011; Solanki et al. 2014; Sayyed et al. 2005). Similar results were also recorded by Venkat et al. and Calvente et al. when dealing with the same bacterial species *Listeria monocytogenes* and *Bacillus thuringiensis* (Calvente et al. 2001; Venkat Kumar et al. 2017).

**FIGURE 3:**
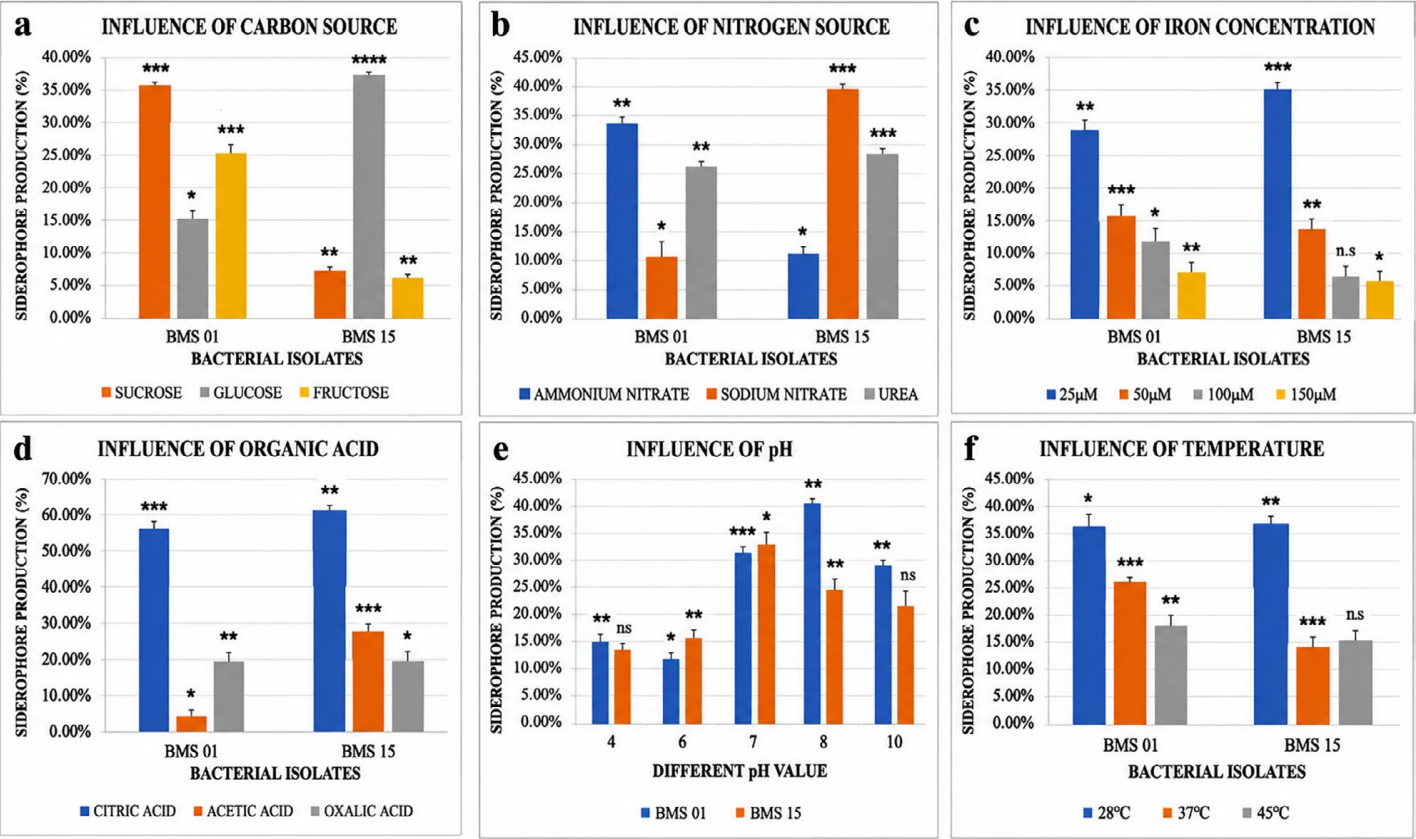
Siderophore production optimisation for bacterial isolates BMS 01 and BMS 15 (a) influence of carbon source (b) influence of nitrogen source (c) influence of iron concentration (d) influence of organic acid (e) influence of pH and (f) influence of temperatures. The *t*‐test was performed for statistical significance. Error bars represent mean ± standard deviation (SD) between three biological replicates. * corresponds to *p* < 0.05, ** corresponds to *p* < 0.01, *** corresponds to *p* < 0.001, *** corresponds to *p* < 0.0001, ‘n.s.’, non‐significant corresponds to *p* > 0.05.

#### 3.4.2 Effect of Different Temperatures

In addition, a steady pH and varied temperatures were used to alter the growing conditions. *Pseudomonas aeruginosa* was shown to have higher siderophore concentration at 27°C than other bacteria, according to earlier investigations on siderophore production and optimisation using the OVAT method (Sharma et al. 2016). When compared to the findings of the last investigation, the bacterial isolates BMS 01 and BMS 15 produced siderophores in high concentrations at 28°C, with 35.48% and 31.73% of the siderophores being produced (Figure 3f).

#### 3.4.3 Effect of Nitrogen

The optimisation process used a variety of nitrogen sources, including urea, sodium nitrate, and ammonium sulfate. According to Figure 3b, both bacterial isolates produced siderophore in almost identical amounts in ammonium sulfate and sodium sulfate as nitrogen sources of about 34.32% by BMS 01 and 37.82% by BMS 15. The outcomes were consistent with isolates of *Rhodotorula* sp. producing siderophores using various nitrogen sources (Calvente et al. 2001). Maximum productivity was demonstrated in the presence of sodium nitrate by *Bacillus thuringiensis* and *Enterobacter soli* (Tailor and Joshi 2012; Louden et al. 2011; Venkat Kumar et al. 2017).

#### 3.4.4 Effect of Different Carbon Sources

The ideal carbon sources that the bacterial strain needs to manufacture the siderophore were examined. Bacterial growth and siderophore synthesis are both increased when carbon sources are added to the growth media. We have chosen sucrose, glucose, and fructose as the main three carbon sources. When impacted by a carbon source like sucrose and glucose, as seen in Figure 3a, both bacterial isolates produced siderophores in increased concentrations. Nevertheless, we contrasted the bacterial isolates with one another. The bacterial isolate was found to produce siderophore at a considerably higher rate that showed an evolutionary connection to the *Bacillus* sp. (Tailor and Joshi 2012; Louden et al. 2011; Simha et al. 2016).

Most of the bacteria that produce siderophores have been identified as belonging to the Bacilli group, based on previous studies of these species. Sucrose, glucose, mannitol, lactose, and xylose were introduced to *Pseudomonas fluorescence*, leading to a reduced rate of siderophore production. This suggests that the organism may have utilised all available carbon sources for growth instead of producing siderophores (Tailor and Joshi 2012; Maindad et al. 2014; Challis 2005; Ghosh et al. 2015). Comparable findings of heightened siderophore production upon the addition of sucrose have been observed in earlier studies involving *Rhodotorula* sp., *Bacillus* sp., and *Enterobacter* sp., as documented in published research by Calvente et al. and Venkat et al. (Tailor and Joshi 2012; Calvente et al. 2001; Venkat Kumar et al. 2017).

#### 3.4.5 Effect of Iron Concentrations+3

The impact of different organic acid and iron concentrations on siderophores production under different culture conditions was the subject of subsequent investigation. Up to a certain point, the increase in iron concentration may have prompted an increase in the production of siderophores, which bind with the available iron and supply it to the cell. Experiments tested iron concentrations ranging from 0 to 150 μM. Siderophore production reached a peak at 25 μM and subsequently declined. This observation is consistent with the hypothesised negative regulation by the Fur (ferric uptake regulator) protein when combined with Fe^2+^, a common regulatory mechanism in many *Bacillus* species, although the specific molecular pathways for these isolates remain to be experimentally validated. Upon reaching 25 μM, the concentration of siderophore fluorescence from *Bacillus* sp., *Enterobacter* sp., and *Pseudomonas* decreased, as previously observed in studies by Tailor and Joshi (2012) and Venkat Kumar et al. (2017). This decline was attributed to the negative regulation of the iron‐regulated gene during transcription (Tailor and Joshi 2012; Ahire et al. 2011; Venkat Kumar et al. 2017) (Figure 3c).

#### 3.4.6 Effect of Organic Acids Source

An uptick in siderophores production was noted when citric acid was present. (Barillot et al. 2012) The results concerning the amounts of siderophore production as a function of organic acid are shown in Figure 3d (Solanki et al. 2014). Sayeed et al. claimed that P. fluorescence and *P. putida* couldn't produce siderophore when exposed to citric acid, but our results show the opposite. (Miethke and Marahiel 2007; Cornish and Page 1995) Both Venkat et al. and Sharma et al. found that supplementing with citric acid increased siderophore production. This may be because our strains showed the greatest efficiency in absorbing citric acid (J. B. Neilands 1995; Ganesapillai and Simha 2015; Ganesapillai, Simha, et al. 2016; Ganesapillai, Singh, and Simha 2016) compared to all of the other organic acids given.

## 4 Conclusion

Iron is necessary for all types of microbes since it is a fundamental component of numerous biological processes. Some microorganisms create siderophores to help them live in iron‐deficient conditions. These microscopic chelators can take up iron from the air or the host, among other sources. *Bacillus safensis* (BMS—01) and *Bacillus tropicus* (BMS—15), two powerful siderophore‐producing rhizobacteria, were effectively isolated from the maize rhizosphere and described in this paper. Their taxonomic position was validated with high sequence identity through molecular identification using 16S rDNA sequencing, which was backed by phylogenetic analysis and GenBank registration (OR136465, OR136385). Environmental and dietary parameters have a considerable impact on siderophore yield, as shown by statistical optimisation utilising a one‐variable‐at‐a‐time (OVAT) technique. Under iron‐limited conditions, both strains showed maximum siderophore synthesis (60%–70% units) at an ideal temperature of 28°C and pH 8.0, with the most efficient carbon and nitrogen sources being glucose and NaNO_3_, respectively. These findings provided a solid foundation for the metabolic behaviour of these strains and were statistically confirmed (*p* < 0.05). The broad‐spectrum iron‐chelating activity detected by CAS assays indicates a great potential for these strains to function as biocontrol agents in iron‐deficient soils, even though this study provides the basic conditions for optimising siderophore synthesis in vitro. These results add to the increasing amount of data that supports the use of Bacillus species as environmentally friendly substitutes for chemical fertilisers. To fully utilise these siderophores' plant growth‐promoting and antagonistic properties in large‐scale agriculture, future studies centered on greenhouse trials and the structural elucidation of these siderophores via LC–MS will be crucial.

## Author Contributions

**Sanober Kalam:** writing – original draft, writing – review and editing, methodology, formal analysis, data curation. **Anita Tilwari:** conceptualization, methodology, formal analysis, supervision, writing – review and editing, writing – original draft, investigation, data curation, resources, visualization.

## Conflicts of Interest

The authors declare no conflicts of interest.

## Data Availability

All data generated or analysed during this study are included in this published article.
